# Morphological and neurophysiological impairment of the nerve in type II macrodactyly

**DOI:** 10.1371/journal.pone.0200183

**Published:** 2018-07-12

**Authors:** Xi Yang, Yongkang Jiang, Shengbo Zhou, Ruiji Guo, Gang Han, Bin Wang

**Affiliations:** Department of Plastic and Reconstructive Surgery, Shanghai 9th People's Hospital, Shanghai Jiaotong University School of Medicine, Shanghai, China; Universidade Federal Fluminense, BRAZIL

## Abstract

**Background:**

Macrodactyly is a congenital malformation characterized by aggressive overgrowth of multiple tissues, including subcutaneous fat, nerves, and bones in digits or limbs. In type II macrodactyly, the peripheral nerve is enlarged; however, the morphological and functional characteristics of the affected peripheral nerves have rarely been evaluated.

**Methods:**

In this research, six macrodactyly patients and three polydactyly patients (control) were studied. Pre-operative sensory nerve action potential and intra-operative nerve action potential tests were performed. The microstructure and ultrastructure of the enlarged nerves were observed and neurofilament (NF) expression was evaluated using immunofluorescent staining.

**Results:**

Axon impairment of the digital nerves originating from the median nerve (MN) was observed. A compensatory reinnervation from the ulnar nerve (UN) was found in two of the six patients, and significant morphological changes were observed in the enlarged nerve. The myelinated nerve fibers decreased, the lamellar structure of the myelin sheath changed, and the density of the NFs of the unmyelinated fibers decreased. There was aberrant distribution of NFs in the macrodactylous nerve tissues. In patients with compensatory UN reinnervation, the number of myelinated and unmyelinated fibers increased to normal levels; however, the diameter of the myelinated fibers apparently decreased.

**Conclusions:**

The morphology and function of the macrodactylous enlarged nerve was impaired in type II macrodactyly patients; however, the unaffected UN partially compensated for the lost function of the affected MN under specific situations. Electrophysiological tests should be performed to determine the function of the affected nerve and surgical treatment for type II macrodactyly could be refined.

## Introduction

**Fig 1:**
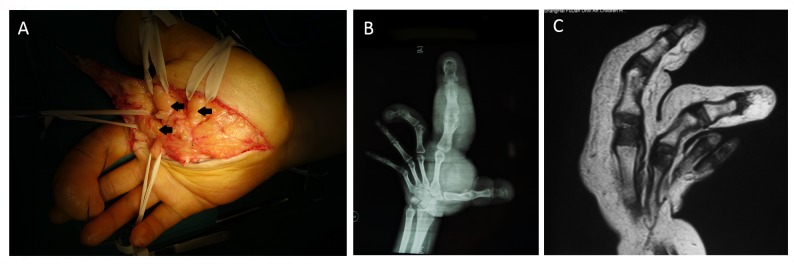
Surgical view and imaging of macrodactyly. (A) Enlargement in the digital nerve in type II macrodactyly (black arrows). (B, C) Adipose overgrowth and bone hypertrophy can be assessed by X-ray and magnetic resonance imaging; however, these techniques cannot accurately display nerve enlargement and functional damage.

Macrodactyly is characterized by excessive growth of multiple tissues including subcutaneous fat, nerve, and bone in digits or limbs, which the patient is tortured by the progression of the disease. PIK3CA somatic mutations were found in these affected tissues [1, 2]. The total incidence rate of macrodactyly is ~1/18000.[3, 4] Type II macrodactyly is characterized by an overgrowth of adipose tissue with peripheral nerve enlargement (Fig 1A) [1].

Clinically, the multiple overgrowth of bone and soft tissue is often assessed by X-rays or magnetic resonance imaging (MRI) (Fig 1B and 1C); however, these methods are not sensitive to detect the nerve enlargement and functional damage. According to previous studies, amputation or debulking by excising a portion of the involved tissue is the main treatment method, and the enlarged nerve is usually excised simultaneously because of the amount of space it occupies[3, 5, 6]; However, nerve function impairment and the possible compensatory pattern of type II macrodactyly are rarely studied for the selection of therapy.

In this study, pre-operative and intra-operative electrophysiological tests were performed on type II macrodactyly patients. The micro- and ultrastructure of the affected nerve was also investigated and neurofilament (NF) expression between the macrodactylous nerve tissue and the normal digital nerve tissue was evaluated. The results provided new information on morphological and neurophysiological impairment of the nerve in type II macrodactyly patients, and might help surgeons make the clinical decision on whether to excise the damaged nerves or preserve the functional compensatory nerve during the surgical procedure.

## Methods

### Patients and nerve samples

Nerve samples from six patients with macrodactyly and control nerve samples from three patients with polydactyly were collected. The patients were recruited from February 2015 to October 2016 and then accepted the reconstructive surgery, and the average age of the patients was 14 ± 4.7 years. Our study was approved by the ethics committee of Shanghai Ninth People’s Hospital, China. The macrodactylous enlarged nerve (MEN) tissues were harvested during debulking surgery in patients with macrodactyly. The nerve tissues excised from the discarded digits of patients with thumb duplication during plastic surgery were used as the normal control. All patients and their parents were informed of the procedures and signed and submitted informed consent forms before the surgery.

The enlarged digital nerves from patients with macrodactyly and the normal digital nerve (NDN) tissues from patients with thumb duplication were isolated and excised during surgery. The nerve tissue was divided into three parts, which was used for hematoxylin and eosin (HE) staining, immunofluorescence staining, and examination by electron microscopy respectively.

### Electromyogram (EMG)

The electrophysiological examination was performed before and during surgery on all patients with macrodactyly. Both the affected side and the healthy side were examined. A Medtronic Keypoint EMG machine (Medtronic Inc., Minneapolis, MN, USA) was used for assessment. In the pre-operation assessment, the recruiting responses of the muscles in the bilateral arm and hand were recorded. Motor and sensory nerve conduction tests were also performed in the median nerves (MNs) and ulnar nerves (UNs) in the affected side. During the intra-operation assessment, the nerve action potentials (NAPs) of the MN to the digital nerve of the index finger and of the UN to the digital nerve of index finger were recorded.

### Paraffin sections and light microscopy

The samples of MEN and the NDN tissues were fixed in 4.0% paraformaldehyde, embedded in paraffin, and sectioned at 4.0-mm intervals, followed by HE staining. The sections were evaluated and recorded using the Eclipse 90i optical microscope (Nikon, Tokyo, Japan).

### Resin sections and electron microscopy

The segments of MEN and the NDN tissues were fixed in 2.0% neutral glutaraldehyde. After washing in phosphate buffer, the samples were fixed for 2.0 h in 1.0% osmium tetroxide at 4.0°C, dehydrated in graded ethyl alcohol, placed in propylene oxide for 10 min, and dried in 70% ethanol containing 3.0% uranyl acetate at 4.0°C. Propylene oxide was incubated into pure resin, polymerized overnight at 37°C, and dried in 60°C. The nerve segments were sectioned at 2.0-mm intervals after resin embedding, and stained with toluidine blue for orientation and observation of the infiltration of lipid droplets using the Eclipse 90i light microscope (Nikon, Tokyo, Japan). The ultrathin sections (100 nm) were then stained with uranyl acetate and examined under an electron microscope (Philips CM-120, Holland) at 80 kV.

### Fluorescence microscopy

After immersion in 4.0% paraformaldehyde for 2.0 h, the nerve samples were embedded in optimal cutting temperature compound and sectioned into 5.0-mm intervals using the Shandon Finesse 325 manual rotary microtome (Thermo Shandon, Cheshire, UK) for immunofluorescence. The primary antibody was anti-NF (1:200, ab7794, mouse monoclonal antibody, Abcam Biotechnology, Cambridge, UK). The secondary antibody was Cy3 AffiniPure goat anti-mouse IgG (1:200, E031610; EarthOx Life Sciences, Millbrae, CA). DAPI (1:500, Invitrogen, Carlsbad, CA) was used for nuclear staining. After antigen retrieval using a microwave, sections were blocked in 3.0% BSA at 37°C for 30 min and incubated in primary antibody at 4.0°C in a humidor. After overnight incubation, nerve tissue sections were washed in PBS for 15–20 min and incubated in secondary antibody at 37°C for 50 min under dark condition, and observed under the Eclipse 90i inverted fluorescence microscope (Cy3 red excitation wavelength 510–560, emission wavelength 590 nm) (Nikon, Tokyo, Japan).

### Statistical analyses

All data were recorded as the mean ± standard deviation (SD), and all statistical analyses were performed using SPSS 19.0 (SPSS Inc., Chicago, IL, USA). The number of myelinated and unmyelinated fibers was compared by electron microscope among three visual fields in different samples. The differences in the number of fibers between the two groups were analyzed by Student’s t-test. P < .05 was considered statistically significant.

## Results

### Neurological dysfunction in the macrodactylous nerve

**Fig 2:**
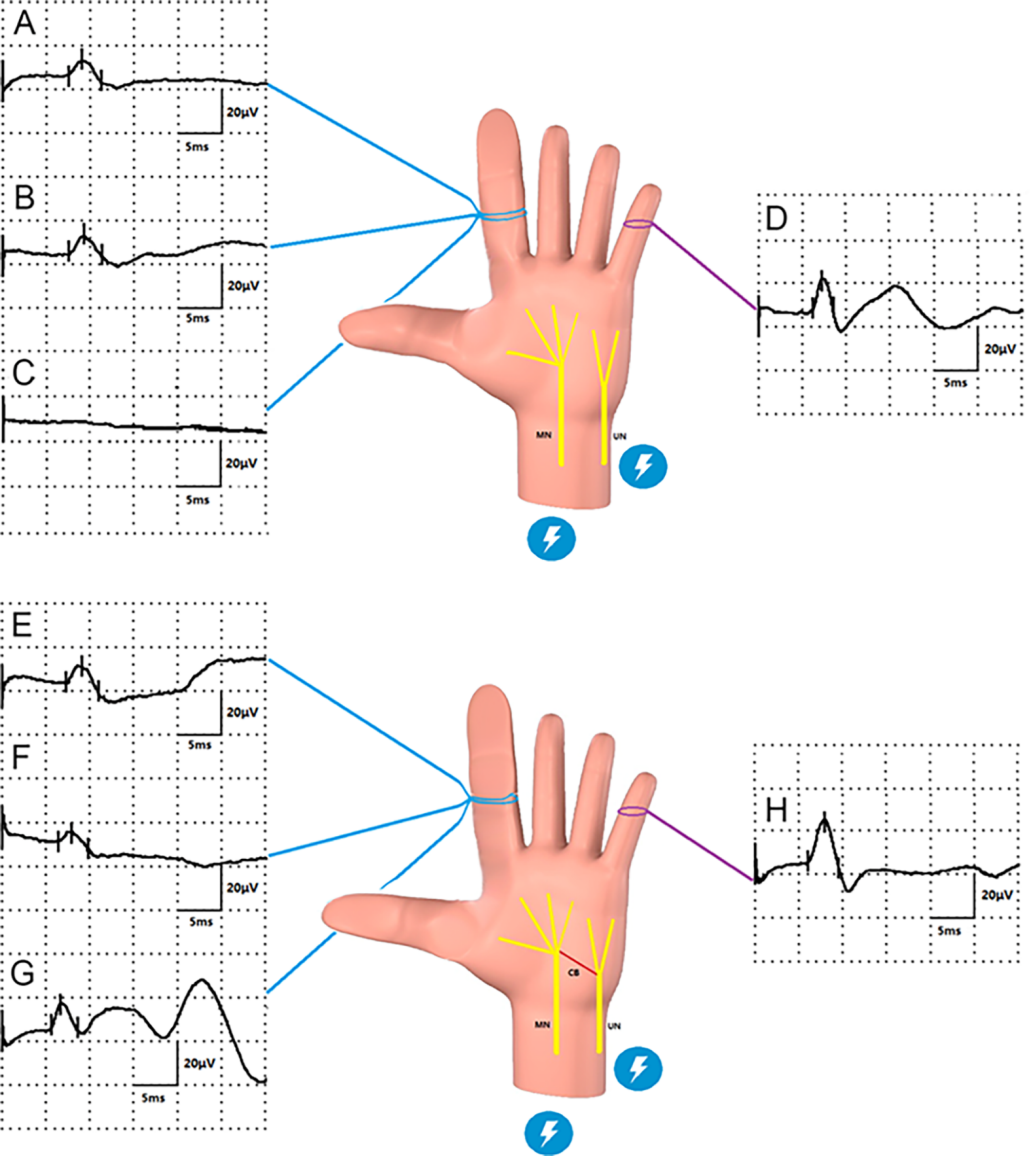
Median nerve damage and compensatory communication branch from ulnar nerve (BA type) in macrodactyly, which was investigated by electromyogram. (A to D) Median nerve damage. In a typical patient without a communication branch from the ulnar nerve to the median nerve, the median nerve was stimulated and showed a prolonged latency and decreased amplitude of electrophysiological signals in the digital nerve of the second finger (A) before surgery and (B) during surgery. Simultaneously, when the median nerve was stimulated, no electrophysiological signals were recorded in the digital nerve of the second finger (C), which suggested no compensatory communication branch from ulnar nerve to median nerve. When the ulnar nerve was stimulated, a normal electrophysiological signal was recorded in the digital nerve of the fifth finger (D). (E to H) Berrettini anastomosis (BA) type. In a typical patient with a communication branch from the ulnar nerve to the median nerve, when the median nerve was stimulated a prolonged latency and decreased amplitude of electrophysiological signals were recorded in the digital nerve of the second fingers before surgery (E) and during surgery (F). Simultaneously, when the ulnar nerve was stimulated, a significant electrophysiological signal was recorded in the digital nerve of the second finger (G), which suggested a compensatory communication branch from the ulnar nerve to the median nerve. We stimulated the ulnar nerve and recorded a normal electrophysiological signal in the digital nerve of the fifth fingers (H).

All patients were examined by electromyography (EMG) (Fig 2). During the pre-operative electrophysiological test, we found that the sensory NAPs amplitude in the index digits significantly decreased with prolonged latency (Fig 2A and 2E). The mean amplitude was 5.0 ±1.2 μV and the mean latency was 7.6 ± 0.8 ms, which suggested axon loss and degeneration of the myelin sheath. In the intra-operative electrophysiological tests, we separated the digital nerves of the index digits by incision and performed the NAPs test directly on the digital nerves of the index finger. We also found that the amplitude significantly decreased in all patients (Fig 2B and 2F). In four of the six patients, the amplitude decreased by 60–80% compared with that of the normal digital nerve. In the remaining two patients, the amplitude decreased by only 30–50% compared with that in the normal digital nerve. We then performed the NAP test from the MN to the affected digital nerve and from the UN to the affected digital nerve. We found that the two patients whose NAP amplitude decreased only by ~50% showed a significant NAP from the UN to the affected digital nerve, which might suggest compensatory UN reinnervation (Fig 2H).

### Fascicle tissue hyperplasia and adipose infiltration in macrodactylous nerve

**Fig 3:**
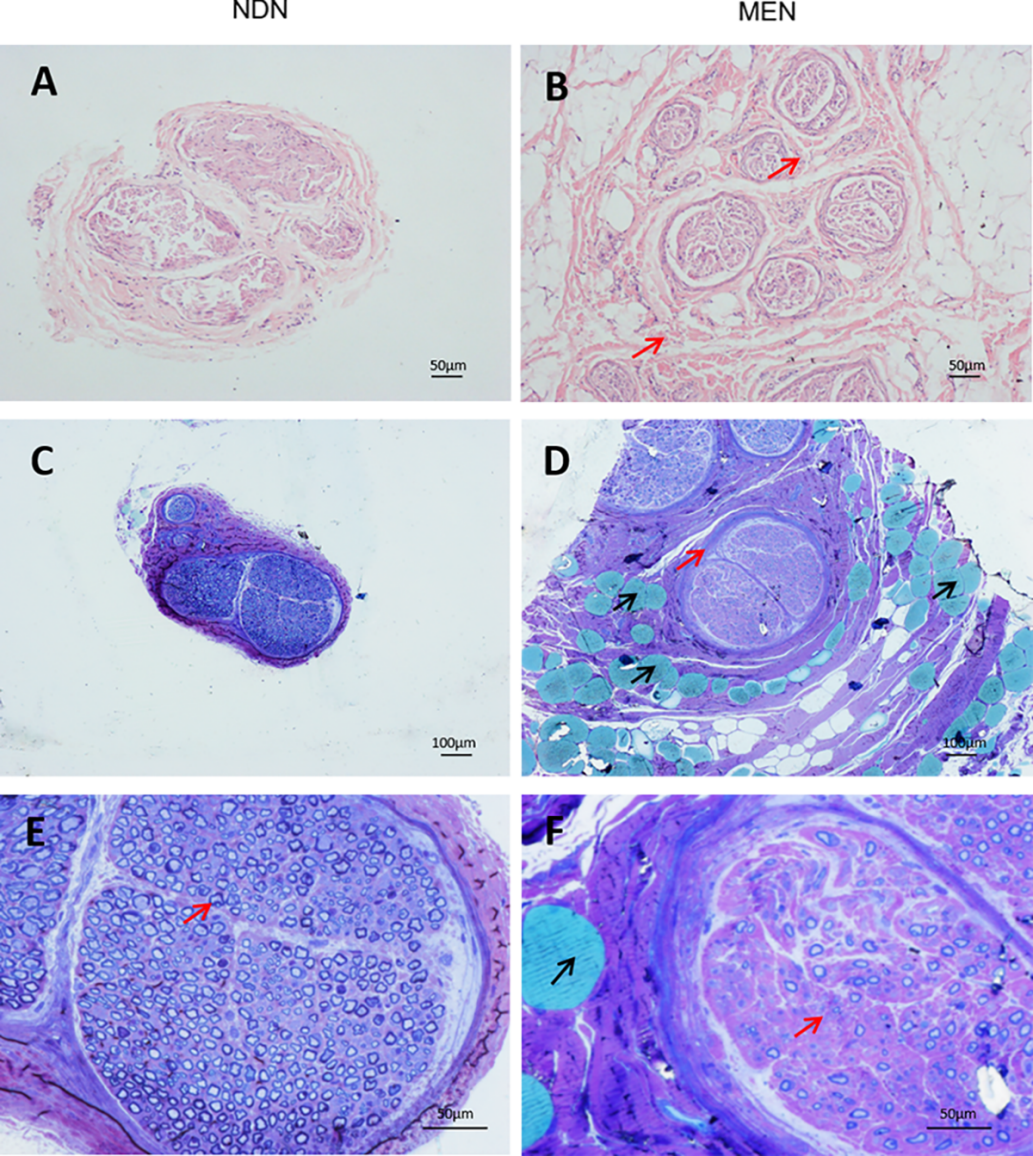
Hematoxylin and eosin (HE) stained paraffin sections and toluidine blue–stained resin sections of the macrodactylous enlarged nerve and the normal digital nerve tissue. (A) HE-stained paraffin sections of the normal digital nerve tissue (scale bars, 50 μm). (B) HE-stained paraffin sections of macrodactylous nerve showed an overgrowth of the nerve bundle, which were separated by thickened epineurium and perineurium (B, red arrows) (scale bars, 50 μm). (C) Toluidine blue–stained resin sections of the normal digital nerve tissue (scale bars, 100 μm). (D) Toluidine blue–stained resin sections of macrodactylous nerves were extensively infiltrated with adipocytes (D, black arrows) and displayed a hyperplasia in interfascicular epineurium (D, red arrows) (scale bars, 100 μm). (E) Intensive distribution of myelinated nerve was found in the normal digital nerve tissue (E, red arrows) (scale bars, 50 μm). (F) A clear decrease in the myelination of the macrodactylous nerve was apparent (F, red arrows). The lipid droplets invaded the interfascicular epineurium and the perineurium (black arrow), but not the endoneurium of the nerve bundle (scale bars, 50 μm).

As shown in the Fig 3A, the stained paraffin sections of the macrodactylous nerve showed an overgrowth of the nerve bundle compared with the normal digital nerve (Fig 3B). The macrodactylous nerve bundles were separated by thickened epineurium and perineurium (triangles), and toluidine blue–stained resin sections (Fig 3C and 3D) showed hyperplasia in the interfascicular epineurium (triangles). As show shown in Fig 3E, myelinated nerve fibers were intensively distributed in the normal digital nerve tissue (triangle), while Fig 3F showed that the density of the myelinated macrodactylous nerve was obviously decreased (triangle), and lipid droplets invaded the interfascicular epineurium and perineurium (arrow), but not the endoneurium of the nerve bundle.

### The structural destruction of the myelinated nerve

**Fig 4:**
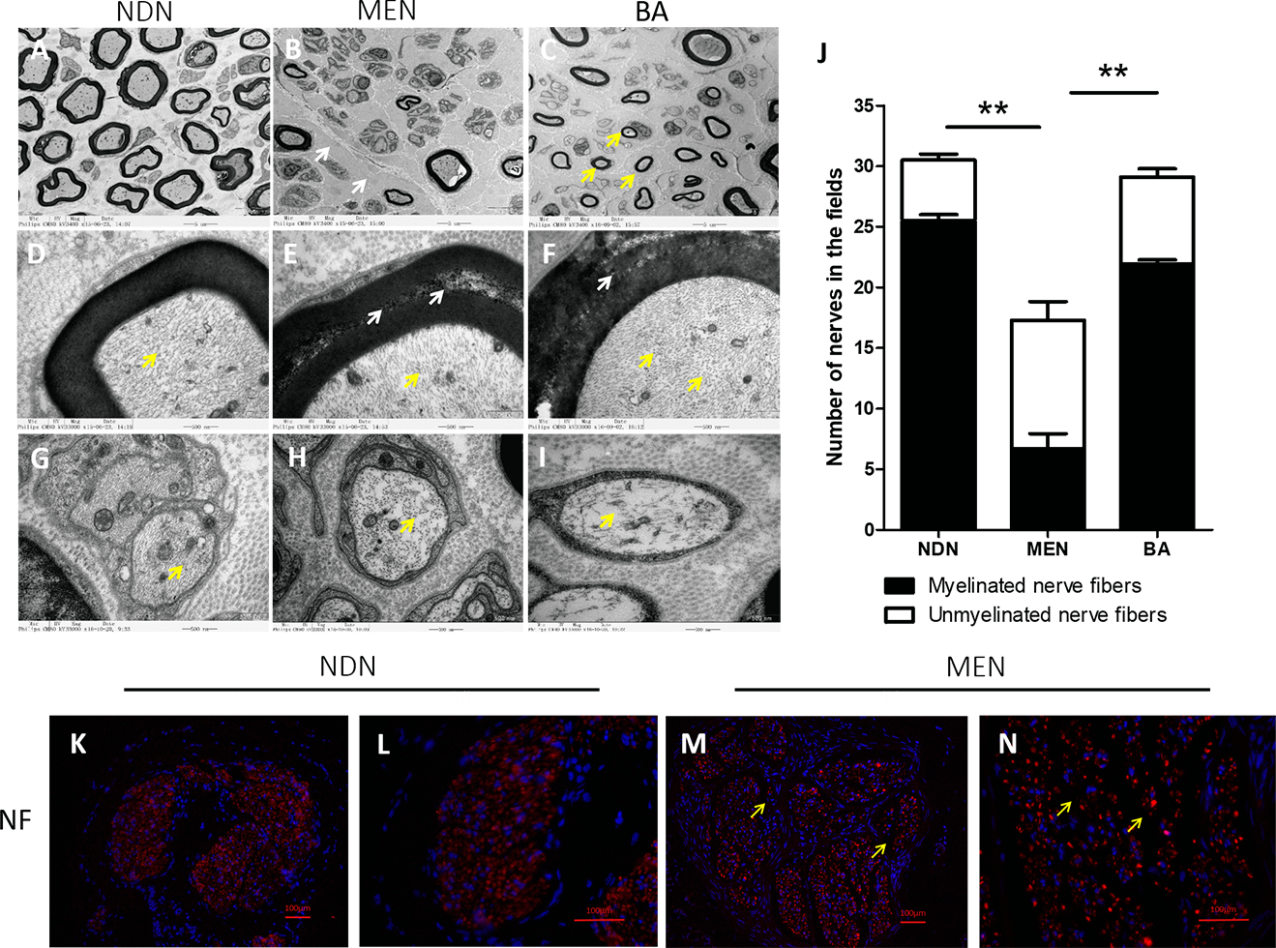
Ultrastructure and immunofluorescent staining of the macrodactylous enlarged nerve (MEN) and normal digital nerve (NDN) tissue. (A) NDN tissue. Myelinated fibers were intensively distributed (scale bars, 5.0 nm). (B) MEN tissue showed a significant decrease in the number of myelinated nerve fibers compared with those in NDN tissue (scale bars, 5.0 nm) and collagen density of the nerve fibers was distinctly increased (B, white arrows) (C) The diameter of the myelinated fiber was apparently reduced in the new regenerative nerve structures (C, yellow arrows), which was found in Berrettini anastomosis (BA) (scale bars, 5.0 nm). (D to F) Compared with NDN tissue, myelin sheath damage was found in both MEN and BA tissues (E, white arrows), while the density of the neurofilament (NF) showed no significant change in the myelinated fiber (D, E, F, yellow arrows) (scale bars, 500 nm). (G to I) The density of the NF of unmyelinated fiber decreased (G, H, I, yellow arrows). (J) Compared with NDN tissue, the number of myelinated and unmyelinated fibers decreased in MEN tissue, while new regenerative nerve structures (BA) increased to nearly normal level (**P < .01). (K to N) Immunofluorescent staining showed dense distribution of NF (red) in NDN tissue (K, L), while the density of NF expression decreased, the fluorescence intensity significantly decreased in some areas (M, N, yellow arrows), which showed an aberrant distribution of NF in the macrodactylous nerve tissue (scale bars, 100 μm).

In MEN tissues, uranyl acetate/lead citrate–stained sections showed a significant decrease in the number of myelinated nerve fibers compared with NDN tissue (Fig 4A and 4B). Compared with NDN tissues, the collagen density of the nerve fibers was distinctly increased (Fig 4B, white arrows). In addition, some of the myelinated fibers lost the normal lamellar structure (Fig 4D, 4E and 4F, white triangles). The density of the NFs showed no significant change in the myelinated fibers (Fig 4D, 4E and 4F, yellow triangles) but apparently decreased in the unmyelinated fiber of MEN tissue (Fig 4G, 4H and 4I, yellow triangles).

Meanwhile, different ultrastructures were found in some of the MEN samples (Fig 4C, 4F and 4I, *Berrettini anastomosis*, *BA*). The number of myelinated and unmyelinated fibers increased to a nearly normal level in BA tissues compared with that in the NDN and MEN tissues (Fig 4J, P < .01), but the diameter of the myelinated fiber was apparently reduced (Fig 4C, yellow arrows), which was considered a characteristic ultrastructure of new regenerative nerves.[7]

NFs in the nerve segments were also observed by fluorescence macroscopy. As shown in Fig 4, intense NF positive staining (red) was found in the normal digital nerve, and as the density of NF expression decreased, the fluorescence intensity also significantly decreased in some areas (Fig 4K, 4L, 4M and 4N, yellow arrows), which showed an aberrant distribution of NF in the macrodactylous nerve tissue.

## Discussion

Previous studies have indicated that resection was the main method for treating the enlarged nerve in type Ⅱ macrodactyly[8]; However, the sensorimotor function of the nerves in the hands is important to the patients, especially in children, and it is difficult to determine whether the enlarged nerve should be retained or excised. In addition, the enlarged nerve often influences the efficiency of defatting surgery[9]. Thus, an effective procedure for hand surgeons or plastic surgeons for diagnosing and treating type Ⅱ macrodactyly was essential.

To evaluate the function of digital nerve, we used a sensory conduction test to assess the function of the affected digital nerve. The results showed an obvious decrease of amplitude, which may imply impairment of axons, and prolonged latency, which may imply degeneration of the myelin sheath (Fig 2); however, the recorded amplitude and latency of the affected nerve in the pre-operative electrophysiological tests could be due to interference resulting from the abnormal proliferation of adipose tissues around the affected nerve, which could hinder the electrical signal recording. Thus, we also performed a NAP test on the affected digital nerve directly during surgery. We found that the decrease in amplitude varied in different individuals, and could be the result of a compensatory mechanism. Thus, we performed the NAP test from MN to the affected digital nerve and from UN to the affected digital nerve. The results demonstrated compensatory ulnar-nerve reinnervation in two of the six patients. It is known that there are several anomalous neural connections between the median and UNs in the upper limb: Martin-Gruber anastomosis (MGA), Marinacci anastomosis (MA), Riche-Cannieu anastomosis (RCA), and Berrettini anastomosis (BA) [10–13]. Among them, BA is a communication in the palmar surface of the hand at the third commissure known as the ramus communicans cumnervi ulnari. It is a neural it consists of a neural connection between the common digital nerves arising from MN and UN. The prevalence of BA is widely variable with rates reported between 4 to 94% [14]. According to our results, it is presumed that the BA could have been the compensatory communication between UN and MN that contributed to the reinnervation in two of the six patients.

For type II macrodactyly, Plaza et al [15] found that the affected nerve showed hyperplasia, intraneural perineurioma–like changes in the enlarged nerve fascicles, and Jonathan et al [1] reported that the enlarged nerve fascicles were separated by endoneurial connective tissue. The thickening of the nerve fascicle tissue and adipose infiltration indicated a neural fibrolipoma–like tumor in type Ⅱ macrodactyly [16, 17]. In our samples, fatty infiltration was distinct in MEN, and lipid droplets invaded the interfascicular epineurium and perineurium; however, fatty infiltration was not involved in the thickened endoneurium of the nerve bundle. Thus, we reasoned that nerve injury in type II macrodactyly patients was not directly caused by fatty infiltration or overgrowth.

We next investigated the ultrastructure of the MEN in type II macrodactyly. Obviously, a decrease in the number of myelinated nerves and loss of the normal lamellar structure could be the reasons for prolonged latency [18, 19], and the decrease in the density of NF in the unmyelinated fiber was consistent with the decrease in amplitude. These MEN ultrastructures were consistent with the results of a previous study [1]. Different ultrastructures were found in some MEN samples. The number of myelinated and unmyelinated fibers in MEN samples was not obviously different from that in normal digital nerve tissue, but the diameter of the myelinated fiber was reduced and was considered as a characteristic ultra-morphology of the new regenerative nerve [7]. This finding was in accordance with the results of EMG regarding the phenomenon of compensatory nerves in type II macrodactyly patients. We speculated that this type of nerve fiber regenerated to compensate the injured digital nerve.

Fluorescence staining showed that NF distribution was aberrant in the nerve fiber in the macrodactylous nerve tissue. Obviously, the decreased expression of NF was located in the unmyelinated nerve fiber. According to a previous study, NF formed the intermediate filament of nerve cells, which played a key role in the neuronal cytoskeleton network during nerve development[20]. Lee et al [21] reported that aberrant organization or assembly of NFs caused diseases arising from selective dysfunction and the degeneration of motor neurons. NF down regulation in the unmyelinated nerve fiber was related to the degeneration of the injured nerve in type II macrodactyly, which could be investigated using electrophysiological tests. These results corresponded to a consistency in hyperplasia and functional damages of the nerve tissue in type II macrodactyly.

**Fig 5:**
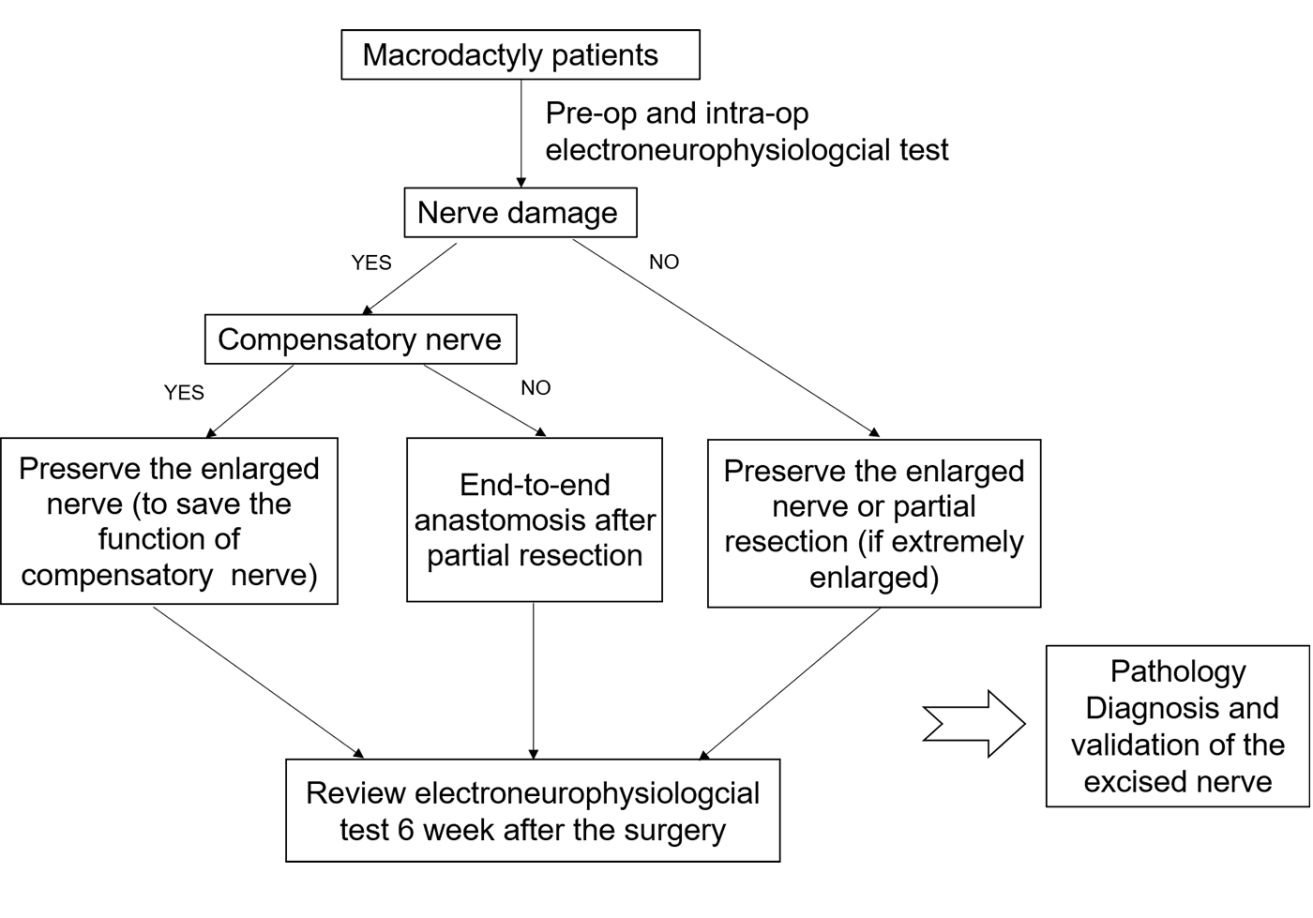
Diagnostic and treated procedures of the enlarged nerve for patients in with type Ⅱ macrodactyly.

In type II macrodactyly, MEN loses its normal morphology and function, and the results of this study showed thickening and a decrease in the function of the nerve. Electrophysiological tests can accurately reflect nerve damage and compensation in type II macrodactyly, which is beneficial to the diagnosis of the disease; therefore, we recommend these diagnostic and treatment protocols for patients with type II macrodactyly. In addition to the consideration and intervention measures of the adipose overgrowth and bone hypertrophy, the involved nerve function of the patient was prudently checked, and we chose different surgical procedures (just for treating the nerve damage and enlargement) according to different circumstances (Fig 5) that would maximize the retention of nerve function and complete the treatment.

In conclusion, we have highlighted that the changes in the micro- and ultra-morphology and the expression of NF in the involved nerve were directly related to nerve thickening and a decrease in nerve function in type II macrodactyly. We also show that electrophysoilogical tests can accurately reflect nerve damage and compensation in type II macrodactyly. We suggest a supplementary diagnostic process and selective surgical treatment for patients with this disease.
